# Herpes Simplex Virus Type 1 Us3 Gene Deletion Influences Toll-like Receptor Responses in Cultured Monocytic Cells

**DOI:** 10.1186/1743-422X-5-140

**Published:** 2008-11-21

**Authors:** Piritta Peri, Riikka K Mattila, Helena Kantola, Eeva Broberg, Heidi S Karttunen, Matti Waris, Tytti Vuorinen, Veijo Hukkanen

**Affiliations:** 1 Department of Virology, University of Turku, Kiinamyllynkatu 13, 20520 Turku, Finland; 2 Department of Medical Microbiology, University of Oulu, Aapistie 5A, 90014 Oulu, Finland

## Abstract

**Background:**

Toll-like receptors have a key role in innate immune response to microbial infection. The toll-like receptor (TLR) family consists of ten identified human TLRs, of which TLR2 and TLR9 have been shown to initiate innate responses to herpes simplex virus type 1 (HSV-1) and TLR3 has been shown to be involved in defence against severe HSV-1 infections of the central nervous system. However, no significant activation of the TLR3 pathways has been observed in wild type HSV-1 infections. In this work, we have studied the TLR responses and effects on TLR gene expression by HSV-1 with Us3 and ICP4 gene deletions, which also subject infected cells to apoptosis in human monocytic (U937) cell cultures.

**Results:**

U937 human monocytic cells were infected with the Us3 and ICP4 deletion herpes simplex virus (d120), its parental virus HSV-1 (KOS), the Us3 deletion virus (R7041), its rescue virus (R7306) or wild type HSV-1 (F). The mRNA expression of TLR2, TLR3, TLR4, TLR9 and type I interferons (IFN) were analyzed by quantitative real-time PCR. The intracellular expression of TLR3 and type I IFN inducible myxovirus resistance protein A (MxA) protein as well as the level of apoptosis were analyzed by flow cytometry. We observed that the mRNA expression of TLR3 and type I IFNs were significantly increased in d120, R7041 and HSV-1 (F)-infected U937 cells. Moreover, the intracellular expression of TLR3 and MxA were significantly increased in d120 and R7041-infected cells. We observed activation of IRF-3 in infections with d120 and R7041. The TLR4 mRNA expression level was significantly decreased in d120 and R7041-infected cells but increased in HSV-1 (KOS)-infected cells in comparison with uninfected cells. No significant difference in TLR2 or TLR9 mRNA expression levels was seen. Both the R7041 and d120 viruses were able to induce apoptosis in U937 cell cultures.

**Conclusion:**

The levels of TLR3 and type I IFN mRNA were increased in d120, R7041 and HSV-1 (F)-infected cells when compared with uninfected cells. Also IRF-3 was activated in cells infected with the Us3 gene deletion viruses d120 and R7041. This is consistent with activation of TLR3 signaling in the cells. The intracellular TLR3 and type I IFN inducible MxA protein levels were increased in d120 and R7041-infected cells but not in cells infected with the corresponding parental or rescue viruses, suggesting that the HSV-1 Us3 gene is involved in control of TLR3 responses in U937 cells.

## Background

Toll-like receptors (TLRs) have an important role in innate immune response to different microbial infections. In humans, the TLR family consists of ten identified TLRs that recognize distinct pathogen-associated molecular patterns (PAMPs) unique for microorganisms [1]. TLRs are differentially distributed within the cell. Cell-surface TLRs bind to lipids and proteins such as microbial lipopeptides (TLR2), lipopolysaccharide (LPS) (TLR4) or flagellin (TLR5) [1]. Intracellular TLRs are localized in endosomes and they bind to dsRNA (TLR3), ssRNA (TLR7 and TLR8) or CpG DNA (TLR9) [1]. Activation of TLRs stimulates different intracellular pathways leading to activation of several transcription factors such as nuclear factor -κB (NF-κB) and IFN regulatory factors (IRFs) [2]. The TLR signaling cascade depends on the cytoplasmic adaptor molecules associated with the intracytoplasmic region of TLRs [3]. One of these adaptor molecules is MyD88, which can associate with all TLRs except for TLR3 [2]. MyD88-dependent pathway in TLR7/9 signaling induces both inflammatory cytokines and type I interferons [4]. MyD88-independent pathway can be stimulated by TLR3 and TLR4, which associate with TIR domain-containing adaptor protein inducing IFN-β (TRIF) leading to IRF-3 or NF-κB activation [2]. The interaction of TRIF and non-canonical IκB kinases IKKε and TANK-binding kinase 1 (TBK1) leads to phosphorylation of IRF-3 by the kinases. IRF-3 translocates to the nucleus and induces several genes such as the IFN-β gene [2]. In addition, TLR3 and TLR4 can activate NF-κB via MyD88-independent signaling pathway leading to production of IFN-β and inflammatory cytokines.

Herpes simplex virus type 1 (HSV-1) causes a variety of infections in humans [5]. This enveloped, double-stranded DNA virus has a relatively large complex genome and it replicates in the nucleus with a replication cycle of approximately 18 hours. HSV-1 remains latent in sensory neurons of its host for life and can reactivate to cause lesions at or near the initial site of infection [5]. Like other herpesviruses, HSV-1 expresses a large number of enzymes involved in metabolism of nucleic acid (e.g. thymidine kinase), DNA synthesis (e.g. DNA helicase/primase) and processing of proteins (e.g. protein kinase). Productive viral infection is accompanied by inevitable cell destruction. HSV-1 has several strategies for evasion of antiviral immune responses of the infected host. These are for example prevention of shut-off of host protein synthesis [6], latent form of infection with no protein expression [7], blocking presentation of antigenic peptides on the cell surface [8,9] and blocking the apoptosis. However, apoptosis is not blocked in HSV-1-infected cells when de novo protein synthesis of HSV-1 is inhibited indicating that the induction of apoptosis is an early event and HSV-1 expresses polypeptides to block apoptosis [10,11]. Infection with HSV-1 lacking either the early protein kinase Us3, immediate-early ICP27 or the ICP4 proteins, results in apoptosis [11-13]. The extent of apoptosis following HSV-1 infection is cell type dependent [11,14,15].

Recent findings suggest that TLRs play a significant role in innate recognition of HSV-1. HSV-1 infection can induce cytokine response via different pathways. The TLR2 pathway has been shown to be involved in the production of inflammatory cytokines. In response to HSV-1 infection, TLR2 mediates cytokine production, which can be detrimental to the host [16]. Moreover, both TLR9-dependent and -independent pathways are involved in IFN-α production in HSV infection [17]. In interferon-producing cells (IPCs) the MyD88-dependent pathway in TLR9 signaling mediates the secretion of type I interferons in response to HSV-1 [18]. Furthermore, defects in the response to HSV-1 via MyD88-dependent pathway can be compensated with MyD88-independent pathway in TLR9 signaling. Mice lacking TLR9 or MyD88 were capable of controlling HSV-1 replication after local infection [18]. Moreover, it has been shown that HSV-1 can be recognized through both TLR2 and TLR9 leading to IL-6 and IL-12 production in bone marrow-derived dendritic cells [19]. Recently, TLR3 was shown to be involved in defence against severe HSV infections of the central nervous system (CNS) [20]. In studies using wild type HSV-1 no significant activation of TLR3 recognition has been observed. However, in studies of apoptosis in HSV-1 infection we have observed different levels of TLR3 gene expression in cells infected with different HSV-1 mutants [21]. This led us to hypothesize, that wild type HSV-1 is able to interfere with TLR3 signaling in infected cells, and possibly has a viral TLR3 inhibitor. HSV can also activate signaling pathways of innate immunity in infected cells, as its UL37 protein is involved in activation of NF-κB through the TRAF6 adaptor protein [22]. The cell death suppressor M45 of mouse cytomegalovirus modulates also activation of TLR3 [23]. Hence it is conceivable that the anti-apoptotic genes of HSV-1 could be involved in modulation of TLR responses. In this work, we have studied the influence of HSV-1 Us3 and ICP4 gene deletions on TLR responses in human monocytic cell cultures.

## Results

### The level of TLR3 mRNA expression was increased in d120 and R7041-infected U937 cells

**Figure 1:**
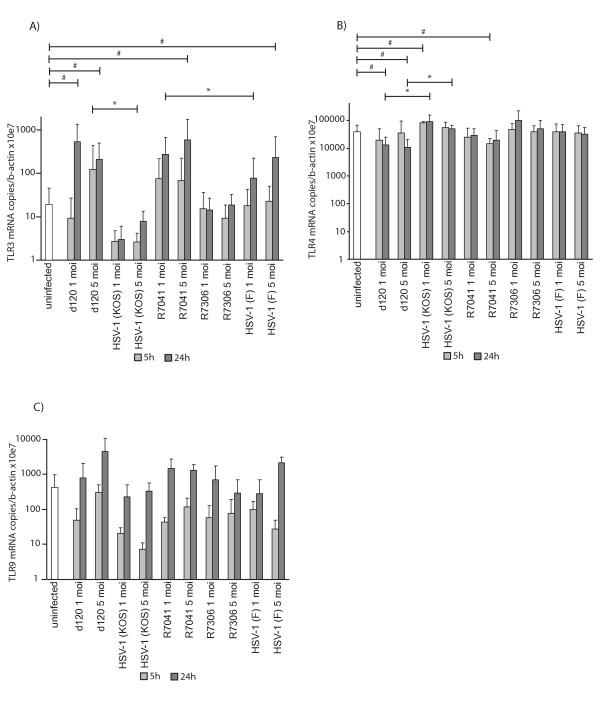
**TLR mRNA expression in infected U937 cells.** The TLR mRNA expression was studied with quantitative real-time PCR at 5h and 24h p.i. A) TLR3 expression. The d120 infection significantly increased the TLR3 expression at 5h p.i. when compared to HSV-1(KOS)-infected cells (5 moi). R7041 infection increased TLR3  expression at 24h p.i. when compared to HSV-1(F)-infected cells (1 moi). B) TLR4 expression. The d120 infection decreased the TLR4 mRNA expression level at 24h p.i. when compared to its parental virus HSV-1(KOS)-infected cells (1 and 5 moi). TLR4 expression level was significantly decreased in d120-infected cells at 24h p.i. (1 and 5 moi) as well as in R7041-infected cells at 5h p.i. (5 moi), but increased in HSV-1(KOS) infection (1 moi) at 5h p.i. when compared to uninfected cells. C) TLR9 expression. No significant differences were seen in TLR9 expression levels. The bars represent the mean level of TLR mRNA expression normalized to β-actin ± standard deviation (SD) from at least three independent experiments. The statistical significances of the differences in TLR copy numbers in comparison with the d120 parental virus HSV-1(KOS) or HSV-1(F) are marked as * (*:p<0.05) and in comparison with uninfected cells as # (#:p<0.05).

To study the effects of HSV-1 infections on TLR gene expression in U937 cells, the mRNA levels of TLR2, TLR3, TLR4 and TLR9 were studied with quantitative real-time PCR at 5 h and 24 h p.i. The d120 infection significantly increased the TLR3 mRNA expression at 5 h p.i. when compared to its parental virus HSV-1 (KOS)-infected cells (5 moi, P = 0.033) (Figure 1A). The R7041 infection increased TLR3 mRNA expression at 24 h p.i. when compared to HSV-1 (F)-infected cells (1 moi, P = 0.021) (Figure 1A). In addition, the TLR3 mRNA expression level was significantly increased in d120 (1 moi, P = 0.009 and 5 moi, P = 0.020), R7041 (5 moi, P = 0.045) and HSV-1 (F)-infected (5 moi, P = 0.043) U937 cells at 24 h p.i. when compared to uninfected cells (Figure 1A). On the contrary, the d120 infection was associated with lowered TLR4 mRNA expression level at 24 h p.i. when compared to its parental virus HSV-1 (KOS)-infected cells (1 moi, P = 0.024 and 5 moi, P = 0.024) (Figure 1B). Also, the TLR4 mRNA expression was significantly decreased in d120-infected cells at 24 h p.i. (1 moi, P = 0.025 and 5 moi, P = 0.015) as well as in R7041-infected cells at 5 h p.i. (5 moi, P = 0.045) but increased in HSV-1 (KOS)-infected (1 moi, P = 0.039) cells when compared to uninfected cells (Figure 1B). No significant difference in TLR2 (data not shown) or TLR9 (Figure 1C) mRNA expression levels between d120, its parental virus HSV-1 (KOS), R7041 and its rescue virus R7306, or HSV-1 (F)-infected and uninfected cells was seen.

### The intracellular expression of TLR3 was increased in d120 and R7041-infected U937 cells

**Figure 2:**
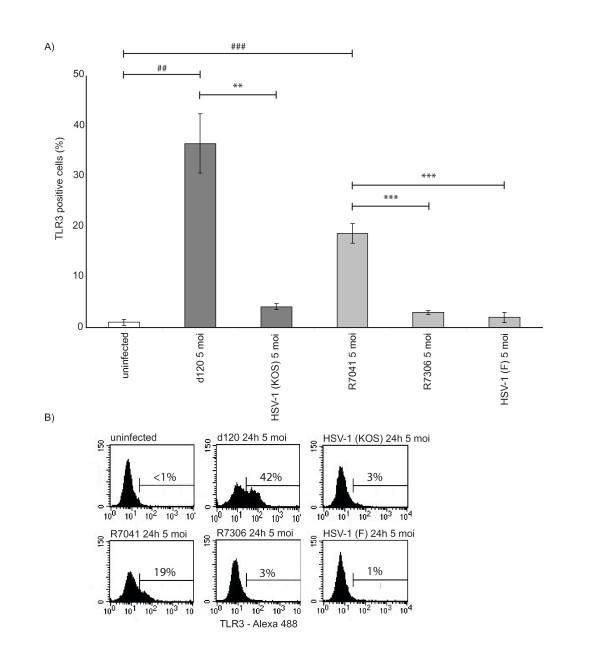
**The intracellular expression of TLR3 was increased in d120 and R7041-infected U937 cells**. A) The U937 cells were infected with d120, its parental virus HSV-1 (KOS), R7041, its rescue virus R7306 or HSV-1 (F) viruses with 5 moi and the level of intracellular TLR3 was studied with flow cytometry at 24 h p.i. The intracellular expression of TLR3 was significantly increased in d120-infected cells when compared to its parental virus HSV-1 (KOS)-infected or uninfected cells. In addition, the TLR3 expression was significantly increased in R7041-infected cells when compared to its rescue virus R7306, HSV-1 (F)-infected or uninfected cells. The bars represent the mean level of TLR3 positive cells ± standard deviation from three independent experiments. The statistical significances of the differences in TLR3 intracellular expression in comparison with the d120 parental virus HSV-1 (KOS) or HSV-1 (F) are marked as * (**:p < 0.01, ***:p < 0.001) and in comparison with uninfected cells are marked as # (##: p < 0.01, ###:p < 0.001). B) Representative flow cytometry histograms showing intracellular expression of TLR3 in uninfected cells or in U937 cells infected with d120, its parental virus HSV-1 (KOS), R7041, its rescue virus R7306 or HSV-1 (F).

To see whether the increased TLR3 mRNA expression correlated with TLR3 protein levels in HSV-1-infected cells, the level of intracellular TLR3 was studied with flow cytometry. The intracellular expression of TLR3 was significantly increased at 24 h p.i. in d120-infected cells when compared to its parental virus HSV-1 (KOS)-infected (P = 0.002) or uninfected (P = 0.001) cells (Figure 2). In addition, the intracellular TLR3 expression was significantly increased in R7041-infected cells when compared to R7306-infected (P < 0.001), HSV-1 (F)-infected (P < 0.001), or uninfected (P < 0.001) cells (Figure 2).

### The d120 and R7041 infections induced the activation of IRF-3 in U937 cells

**Figure 3:**
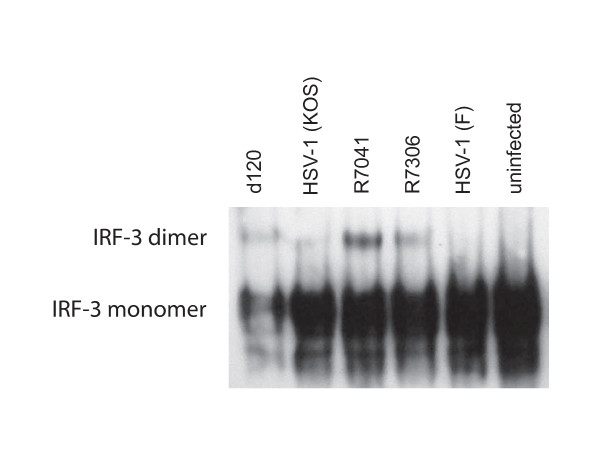
**The d120 and R7041 virus infections induced activation of IRF-3 in U937 cells**. The U937 cells were infected with d120, its parental virus HSV-1 (KOS), R7041, its rescue virus R7306 or HSV-1 (F) viruses with 5 moi and the activation of IRF-3 was studied with a native western blot at 5 h p.i. The d120 and R7041 infections, unlike the parental virus infections, induced the dimerization of IRF-3 at 5 h p.i.

We studied whether the increased TLR3 expression correlated with activation of the downstream factors of the signaling pathway. We could observe dimerization of IRF-3 in infections with d120 and R7041 at 5 h p.i., but not in infections with the parental viruses (Figure 3). Only weak activation of the IRF-3 was seen in infections with the rescue virus R7306 at 5 h p.i. (Figure 3).

### The level of type I IFN mRNA expression was increased in d120 and R7041-infected U937 cells

**Figure 4:**
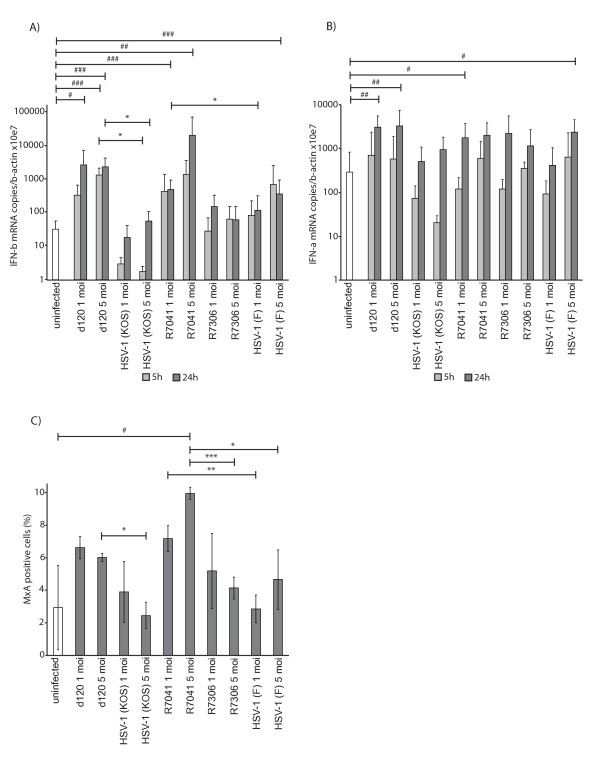
**The expression of type I IFN mRNA and MxA protein in HSV-infected cells.** A) IFN-β mRNA expression. The d120 infection increased the IFN-β expression when compared to HSV-1(KOS)-infection (5 moi). R7041 infection increased the IFN-β expression when compared to HSV-1(F)-infected cells at 24h p.i. (1 moi). B) IFN-α mRNA expression. The IFN-α expression was increased at 24h p.i. in d120 (1 and 5 moi), R7041 (1 moi) and in HSV-1(F)-infected cells (5 moi) when compared to uninfected cells. The bars (A-B) represent the means of IFN-α or IFN-β mRNA normalized to β-actin  ± SD from at least three independent experiments. C) Flow cytometric analysis of intracellular MxA at 24h p.i. The expression of IFN-inducible MxA protein was increased in d120-infected cells when compared to HSV-1(KOS)-infected cells (5 moi). MxA expression was increased in R7041-infected cells when compared to the rescue virus R7306 (5 moi), HSV-1(F)-infected (1 and 5 moi) or uninfected cells. The bars represent the mean level of MxA expression ± SD from three independent experiments. The significances of the differences in comparison of the deletion viruses versus parental viruses are marked as * (*:p<0.05, **:p<0.01, ***:p<0.001) and in comparison with uninfected cells as # (#:p<0.05, ##:p<0.01, ###:p<0.001).

To study if the Us3 deletion virus infections induced further production of type I IFNs, the IFN-α and IFN-β mRNA expression levels were studied with quantitative real-time PCR at 5 h and 24 h p.i. The d120 infection significantly increased the IFN-β mRNA expression when compared to its parental virus HSV-1 (KOS)-infected cells at 5 h and 24 h p.i. (5 moi, P = 0.017 and 5 moi, P = 0.024, respectively) (Figure 4A). The R7041 infection increased the IFN-β mRNA expression when compared to HSV1 (F)-infected cells at 24 h p.i. (1 moi, P = 0.016) (Figure 4A). Also, the IFN-β mRNA expression level was significantly increased at 5 h p.i. in d120-infected cells (5 moi, P < 0.001) and at 24 h p.i in d120 (1 moi, P = 0.015 and 5 moi, P < 0.001), R7041 (1 moi, P < 0.001 and 5 moi, P = 0.007) and in HSV-1 (F)-infected (5 moi, P < 0.001) cells when compared to uninfected cells (Figure 4A). Moreover, the IFN-α mRNA expression level was significantly increased at 24 h p.i. in d120 (1 moi, P = 0.001 and 5 moi, P = 0.003), in R7041- (1 moi, P = 0.031) and in HSV-1 (F)-infected (5 moi, P = 0.025) cells when compared to uninfected cells (Figure 4B).

### The intracellular expression of MxA was increased in d120 and R7041-infected U937 cells

To see whether the increased IFN-β mRNA was also translated to functional IFN-β, we observed the effects on the intracellular IFN-induced MxA protein expression in infected U937 cells. The d120 infection increased the intracellular expression of MxA when compared to its parental virus HSV-1 (KOS)-infected cells (5 moi, P = 0.012) (Figure 4C). The R7041 infection significantly increased the intracellular expression of MxA when compared to its rescue virus R7306 (5 moi, P < 0.001), HSV-1 (F)-infected (1 moi, P = 0.003 and 5 moi, P = 0.033) or uninfected (5 moi, P = 0.040) cells at 24 h p.i. (Figure 4C).

To study further the TLR signaling pathways, the mRNA expression of MyD88, TRIF and IRF-3 were studied with quantitative real-time PCR at 5 h and 24 h p.i. There were no statistical differences between HSV-1 wild type-, Us3 deletion virus-infected or uninfected cells in the MyD88, TRIF or IRF-3 mRNA expression levels (data not shown).

### The amount of apoptosis was increased in d120 and R7041-infected U937 cells

**Figure 5:**
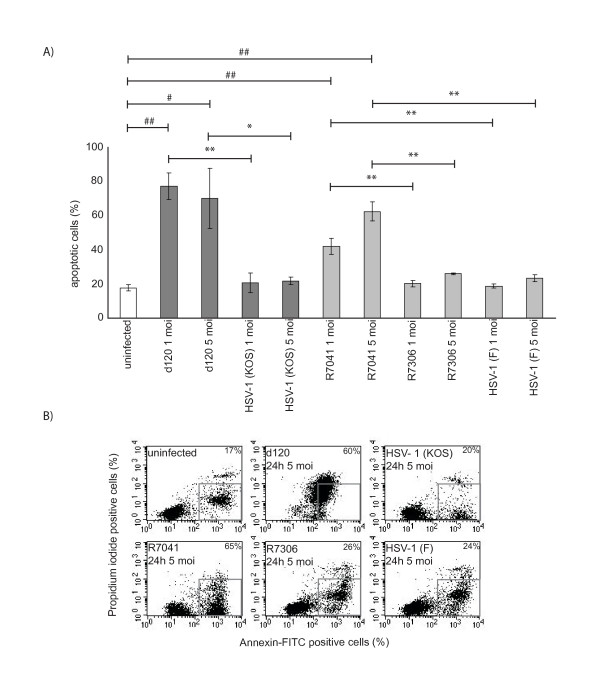
**The d120 and R7041 virus infections induced apoptosis in U937 cells.** The percentage of apoptotic cells was analyzed at 24h p.i. with Annexin V/propidium iodide double staining and flow cytometry. A) Percentage of apoptotic U937 cells. The level of apoptosis was significantly increased in d120-infected cells when compared to its parental virus HSV-1(KOS)-infected (1 and 5 moi) cells. Also, the level of apoptosis was significantly increased in R7041-infected cells when compared to its rescue virus R7306 (1 and 5 moi) and HSV-1(F)-infected (1 and 5 moi) cells. The bars represent the mean level of apoptotic cells ± SD from three independent experiments. The statistical significances of the differences in the level of apoptosis in comparison of the deletion viruses versus the parental viruses are marked as * (*:p<0.05, **:p<0.01) and in comparison with uninfected cells as # (#:p<0.05, ##:p<0.01). B) Representative flow cytometry dot plots showing Annexin V/propidium iodide double staining of uninfected, d120, its parental virus HSV-1(KOS), R7041, its rescue virus R7306 and HSV-1(F)-infected cells. The apoptotic cell population with positive staining for Annexin V and negative staining for propidium iodide is gated from the dot plots.

The amount of apoptotic cells was analyzed at 24 h p.i. with Annexin V/propidium iodide double staining and flow cytometry. The level of apoptosis was significantly increased in d120-infected U937 cells when compared to its parental virus HSV-1 (KOS)-infected cells (1 moi, P = 0.003 and 5 moi, P = 0.040) (Figure 5). The level of apoptosis was also significantly increased in R7041-infected cells when compared to its rescue virus R7306 (1 moi, P = 0.007 and 5 moi, P = 0.007) and HSV-1 (F)-infected cells (1 moi, P = 0.010 and 5 moi, P = 0.003) (Figure 5). In addition, the proportion of apoptotic cells was significantly increased in the d120 (1 moi, P = 0.004 and 5 moi, P = 0.034) and R7041 infections (1 moi, P = 0.006 and 5 moi, P = 0.003) when compared to uninfected cells (Figure 5).

## Discussion

In this work we have shown that the expression level of both the TLR3 mRNA and protein were significantly elevated in HSV-1 Us3 deletion virus-infected U937 cells. Moreover, the Us3 deletion viruses induced strong activation of IRF-3 and type I IFN mRNA expression. We have also shown in the present study, that the expression of interferon-induced MxA protein was increased in d120 and R7041-infected cells showing that functional type I IFN was produced. These findings suggest that HSV-1 viruses with deletions in Us3 or both in Us3 and ICP4 genes may not be able to downregulate the HSV-1 infection-induced TLR3-mediated response in infected U937 cells.

The TLR3 pathway plays a role in the clearance of certain virus infections and survival of the infected organism. On the other hand, strong or sustained TLR3 signaling may be harmful for the host. To control the TLR3-mediated response, cells have several mechanisms to negatively regulate the TLR3 signaling [24]. For example endogenous sterile α- and armadillo-motif-containing protein (SARM) is a negative cellular regulator of NF-κB and IRF activation [25]. Beside the endogenous inhibitors, TLR3-mediated signaling can be inhibited by viral inhibitors. Viral inhibitors of the TLR3 pathway have been described, encoded by e.g. vaccinia virus [26,27], hepatitis A virus [28] and hepatitis C virus [29-31]. The M45 cell death suppressor of mouse cytomegalovirus also may modulate the activation of TLR3 [23]. Thus far, TLR3 pathway inhibitor of HSV-1 has not been reported. Since the TLR3 levels and IFN responses increased in the infections with Us3 or Us3 and ICP4 deletion viruses, it could be conceivable, that US3 and/or ICP4 might act as inhibitors of TLR3-mediated signaling. In further studies we will address this question at the molecular level.

Besides induction of cytokine secretion, TLR-signaling cascades have been reported to result in cell death. Recently, Salaun et al. reported that TLR3 can directly trigger apoptosis in human cancer cells [32]. Synthetic dsRNA both induced apoptosis and blocked the proliferation of breast cancer cells in a TLR3 and TRIF-dependent manner. In addition, type I IFN signaling was shown to be required for TLR3-triggered cytotoxicity, although it was insufficient to induce apoptosis by itself [32]. Moreover, Salaun et al. showed that the synthetic dsRNA-triggered apoptosis was reduced with broad caspase inhibitor treatment, indicating that caspases are involved in TLR3-mediated apoptosis [32]. In the other study, Salaun et al. demonstrated that human melanoma cells were able to express functional TLR3 protein and that the combination of synthetic dsRNA and IFN-α activated caspases and affected apoptosis regulatory molecules [33].

As shown in our study, the d120 and R7041 infections led to apoptosis in U937 cells. It is possible that the HSV-1 without Us3 and ICP4 genes may in part facilitate apoptosis in a TLR3-mediated manner. The exact roles of TLR3 and caspases in HSV-1-induced apoptosis in U937 cells should be further studied. Apart from the described antiapoptotic function of Us3 of HSV-1 [12,34-38], Us3 has been reported to play roles in the transit of capsids from nuclei to cytoplasm and in the phosphorylation of histone deacetylase 1 (HDAC1) and HDAC2 [39,40]. It may also have more functions, which have not yet been described. It is possible, that other cytoplasmic factors, such as DNA-dependent activator of IFN-regulatory factors (DAI) [41], could be involved in the induction of type I IFN genes. The HSV-1-induced expression of TLR3 and type I IFN might be cell type specific. We have also tested human B-lymphoblast cell line (RPMI-8226) for TLR and IFN mRNA expression, but no significant difference was seen between HSV-1-infected and uninfected cells. We have also measured the infectivity of studied viruses and there was no significant difference between HSV-1 (F) and the deletion viruses in U937 cells.

## Conclusion

In the present study, we show that the HSV-1 infection increased the mRNA expression of TLR3 and type I IFNs in U937 cells. However, the expression of intracellular TLR3 or type I IFN inducible MxA protein, and also activation of IRF-3 were increased only in cells infected with Us3 or both Us3 and ICP4 deletion viruses. This suggests that the Us3 interferes with TLR3 recognition and subsequent induction of MxA protein by type I IFN. We conclude, that based on the results of the present study, the Us3 deletion influences the TLR responses to HSV-1 in monocytic cell cultures.

## Methods

### Viruses and cell cultures

Human monocytic (U937) cell cultures were infected with wild type herpes simplex virus type 1 (HSV-1) (F), the Us3 deletion virus (R7041) [42], its repair virus (R7306) [42], the Us3 and ICP4 deletion virus (d120) [43], or its backbone virus (KOS) [43] at a multiplicity (moi) of infection of 1 and 5, and the infections proceeded at 37°C in RPMI 1640 medium with 10% fetal calf serum (FCS). Cells and culture media were collected at early and late time point of infection (5 and 24 h, respectively). The R7041 and R7306 viruses were generously provided by Dr. Bernard Roizman (University of Chicago), the d120 virus was a kind gift from Dr. Neal DeLuca (University of Pittsburgh) and the HSV-1 (KOS) virus was a kind gift from Dr. William Goins (University of Pittsburgh). U937 cells (American Type Culture Collection) were cultured at the concentration of 1 × 10^6 ^ml^-1 ^in RPMI 1640 medium containing 10% FCS, 1% glutamine and gentamicin and were maintained at 37°C in 5% CO_2_.

### RNA extraction, production of cDNA and quantitative real-time PCR

The RNA was extracted from 2 × 10^6 ^of U937 cells at 5 and 24 h p.i. Cells were washed with sterile PBS and the RNA was extracted using the TRIZOL reagent (Invitrogen, Carlsbad, CA, USA) or TriPure reagent (Roche, Basel, Switzerland). The cDNA was synthesized using M-MLV reverse transcriptase (Promega, Madison, USA) and random hexamer primers for 1 h at 37°C. Quantitative real-time PCR was performed in Rotor-Gene™ 6000 instrument (Corbett Life Science, Sydney, Australia) using QuantiTect™ SYBR^® ^Green system (Qiagen, Hilden, Germany), forward and reverse primers for each target of interest (table 1), and 2 μl of the cDNA or diluted PCR standard. The PCR protocol consisted of an initial incubation for 15 min at 95°C followed by PCR cycling using a three step cycle at 95°C for 15 sec, at 60°C (or 55°C for MyD88) for 30 sec and at 72°C for 45 sec for a total of 40 cycles. The cellular β-actin mRNA was studied by quantitative real-time PCR as a control for cellular mRNA changes during the HSV infections as described previously [44]. External standards representing nucleotides 233–514 (TLR2), 41–334 (TLR3), 122–457 (TLR4), 3030–3446 (TLR9), 489–659 (IFN-α), 92–497 (IFN-β), 513–1022 (MyD88), 1008–1490 (IRF-3), and 1338–1887 (TRIF) of each gene were constructed from the cDNA transcripts of RNA isolated from cultures of stimulated human peripheral blood mononuclear cells or of U937 cells. The copy numbers of the standards were calculated as described earlier [45]. A dilution series of standards of 10^1 ^to 10^8 ^copies per reaction were used for each PCR run. The PCR results represent three to ten separate experiments.

**Table 1:** Primers for real-time PCR.

Primer		Sequence (5'> 3')
TLR2	Forward	CAGGGCTCACAGAAGCTGTAA
	Reverse	GCCCAGGGAAGAAAAAGAATC

TLR3	Forward	TAGCAGTCATCCAACAGAATCAT
	Reverse	AATCTTCTGAGTTGATTATGGGTAA

TLR4	Forward	ACACAGAAGAGCTGGCATGA
	Reverse	GGTTGTCGGGGATTTTGTAG

TLR9	Forward	CTTCCCTGTAGCTGCTGTCC
	Reverse	CCTGCACCAGGAGAGACAG

IFN-α(1/13)	Forward	TGGCTGTGAAGAAATACTTCCG
	Reverse	TGTTTTCATGTTGGACCAGATG

IFN-β	Forward	TCTCCACGACAGCTCTTTCCA
	Reverse	ACACTGACAATTGCTGCTTCTTTG

MyD88	Forward	TGGCACCTGTGTCTGGTCTA
	Reverse	ACATTCCTTGCTCTGCAGGT

IRF-3	Forward	GTTCTGTGTGGGGGAGTCAT
	Reverse	CTGTTGGAAATGTGCAGGTC

TRIF	Forward	CCTCCTCCTCCTCCTTCATC
	Reverse	GCGTGGAGGATCACAAAGTT

### Determination of intracellular TLR3

For TLR3 intracellular staining, 1 × 10^6 ^of U937 cells were infected as described above. The cells were collected at 24 h p.i. and fixed with 3% paraformaldehyde for 15 min and permeabilized with 0.1% TritonX-100 for 5 min. Permeabilized cells were washed with 0.5% bovine serum albumin (BSA) in phosphate buffered saline (PBS) and stained with monoclonal antibody to TLR3 (Axxora, San Diego, CA, USA) at the dilution 1:100 and with Alexa Fluor 488 goat anti-mouse IgG (Invitrogen Molecular Probes, Carlsbad, CA, USA) at the dilution 1:200. For analysis, 10 000 cells were collected with FACScan^® ^flow cytometer (Becton Dickinson Biosciences, San Jose, CA, USA) and analyzed with Cell Quest™ software. The flow cytometric data represent three separate experiments.

### Determination of the intracellular MxA protein

For MxA intracellular staining, 1 × 10^6 ^of U937 cells were infected as described above. The U937 cells were stained as described earlier [46]. Briefly, the cells were fixed with paraformaldehyde and permeabilized with TritonX-100. The intracellular MxA protein was stained with a rabbit anti-MxA serum [47] at the dilution 1:1000. Fluorescein-conjugated goat F(ab')_2 _anti-rabbit IgG (Caltag Laboratories, South San Francisco, CA, USA) was used as a secondary antibody at the dilution 1:670. For analysis, 10 000 cells were collected with FACScan^® ^flow cytometer (Becton Dickinson) and analyzed with Cell Quest™ software. The flow cytometric data represent three separate experiments.

### Western blot for the detection of IRF-3 monomer and dimer

To detect the monomeric and dimerized IRF-3, 2 × 10^6 ^of U937 cells were infected as described above. Cells were collected at 5 h and 24 h p.i. and the total protein was extracted with the ProteoJET™ Mammalian Cell Lysis Reagent (Fermentas, Burlington, Canada). The cell samples were electrophoresed with NuPAGE Electrophoresis System at 150 V for 2.5 h on a 10% polyacrylamide gel in Tris-Glycine native running buffer (25 mM Tris base, 192 mM Glycine, pH 8.3). Gels were transferred to Hybond ECL nitrocellulose membrane (Amersham Biosciences, NJ, USA) using NuPAGE Transfer buffer (25 mM Bicine, 25 mM Bis-Tris, 1 mM EDTA, pH 7.2) at 30 V for 75 min. Blots were blocked with 5% milk-TBS-T. The IRF-3 monomer and dimer were detected with IRF-3 polyclonal antibody (Santa Cruz Biotechnology Inc., Santa Cruz, CA, USA) at the dilution of 1:1000 and with secondary HRP-conjugated goat anti-rabbit antibody (Jackson ImmunoResearch Laboratories Inc., West Grove, PA, USA) at the dilution of 1:3300. The equal protein loading was confirmed by blotting for GAPDH from the same samples using a denaturing gel (data not shown).

### Determination of apoptosis

The number of apoptotic cells was measured with flow cytometry at the time points of 5 h and 24 h p.i. The double staining of Annexin V/propidium iodide was used to differentiate between apoptotic and necrotic cells. The U937 cells were washed with PBS and stained with early apoptosis marker Annexin-V-Fluos (Becton Dickinson Biosciences, San Jose, CA, USA) at the dilution of 1:100 and propidium iodide with the concentration of 50 μg ml^-1 ^in Hepes buffer at +4°C for 15 min. For analysis, 10 000 cells were collected with FACScan^® ^flow cytometer (Becton Dickinson). The apoptotic cell population with positive staining for Annexin V and negative staining for propidium iodide was analyzed with Cell Quest™ software. The flow cytometric data represent three separate experiments.

### Statistical analyses

The statistical analyses were performed either with the non-parametric one-way analysis of variance and Wilcoxon scores (quantitative real-time PCR analyses) or with Student's t-test (flow cytometric analyses). Values from d120-, its parental virus HSV-1 (KOS), R7041-, its rescue virus R7306 or HSV-1 (F)-infected cells were compared pairwise with corresponding values of uninfected cells. In addition, values from d120-infected cells were compared pairwise with corresponding values of its parental virus HSV-1 (KOS)-infected cells and values from R7041-infected cells were compared pairwise with values of its rescue virus R7306 or HSV-1 (F)-infected cells. Values of P < 0.05 were considered statistically significant.

## Competing interests

The authors declare that they have no competing interests.

## Authors' contributions

PP participated in the design of the study, carried out the experimental infections and the statistical analyses, and drafted the manuscript. RKM participated in the PCR and protein assays. HK participated in the PCR assays. EB, HSK, MW and TV participated in the design and coordination of the study. VH conceived of the study, participated in its design and coordination, and helped to draft the manuscript. All authors have read and approved the final manuscript.
